# Chronic Oral Exposure to Bisphenol A Results in a Nonmonotonic Dose Response in Mammary Carcinogenesis and Metastasis in MMTV-erbB2 Mice

**DOI:** 10.1289/ehp.1103850

**Published:** 2011-10-12

**Authors:** Sarah Jenkins, Jun Wang, Isam Eltoum, Renee Desmond, Coral A. Lamartiniere

**Affiliations:** 1 Department of Pharmacology and Toxicology,; 2 Department of Anatomic Pathology,; 3 Comprehensive Cancer Center, and; 4 Department of Medicine, Biostatistics Unit, Division of Preventative Medicine, University of Alabama at Birmingham, Birmingham, Alabama, USA

**Keywords:** apoptosis, bisphenol A, BPA, mammary gland, MMTV-erbB2 mice, oral exposure, tumorigenesis

## Abstract

Background: Bisphenol A (BPA) is a synthetic compound used to produce plastics and epoxy resins. BPA can leach from these products in appreciable amounts, resulting in nearly ubiquitous daily exposure to humans. Whether BPA is harmful to humans, especially when administered orally in concentrations relevant to humans, is a topic of debate.

Objectives: In this study, we investigated the role of chronic oral exposure to BPA during adulthood on mammary carcinogenesis by using a transgenic mouse model that spontaneously develops tumors through overexpression of wild-type erbB2 [mouse mammary tumor virus (MMTV)-erbB2].

Methods: MMTV-erbB2 mice were exposed to 0, 2.5, 25, 250, or 2,500 µg BPA/L drinking water from 56 until 112 days of age (for mechanism of action) or 252 days of age (for tumorigenesis). Cellular and molecular mechanisms of BPA action in the mammary gland were investigated via immunohistochemistry and immunoblotting.

Results: Only low doses of BPA significantly decreased tumor latency and increased tumor multiplicity, tumor burden, and the incidence of metastasis. All BPA doses significantly increased the cell proliferation index, but only the higher doses also increased the apoptotic index in the mammary gland. At the molecular level, 25 µg BPA/L, but not 2,500 µg BPA/L, increased phosphorylation of erbB2, erbB3, insulin-like growth factor 1 receptor, and Akt in the mammary gland.

Discussion: Low, but not high, BPA doses significantly accelerated mammary tumorigenesis and metastasis in MMTV-erbB2 mice. The combined ratio of cell proliferation and apoptosis indices and alterations in protein expression best predicted the ability of each dose of BPA to alter tumorigenesis in this model.

The environmental contaminant bisphenol A (BPA) is used in the production of polycarbonate plastics and epoxy resins. These products are used in many common consumer goods, such as food and drink containers, the lacquer lining of canned foods and drinks, infant formula bottles, office water coolers, sales receipts, and some dental sealants. Several recent studies (Brede et al. 2003; Howdeshell et al. 2003; Joskow et al. 2006; Kang et al. 2003) have found that BPA can leach from these products during normal use. Recent reports show that ≥ 90% of the populations studied had detectable concentrations of BPA metabolites in the urine (Calafat et al. 2005, 2008; Wolff et al. 2007). These studies have reported mean values of total urinary BPA to be 1–3 µg BPA/L urine, with values ranging from below the level of detection to > 100 µg BPA/L urine. Estimates of daily BPA intake based on models of BPA pharmacokinetics or exposure sources suggest that the mean adult is exposed to 0.4–1.4 µg BPA/kg body weight (BW), and the 95th percentile of exposure does not exceed 1.5–4.2 µg BPA/kg BW per day [Food and Agricultural Organization/World Health Organization (FAO/WHO) 2010; Lakind and Naiman 2008]. However, estimations of daily human exposure to BPA is a controversial subject that has recently been disputed (Taylor et al. 2011; Vandenberg et al. 2007, 2010).

One concern over BPA stems from its classification as a xenoestrogen. BPA is reported to bind to the estrogen receptors (ER), albeit with an affinity several orders of magnitude less than estradiol, and induce downstream transcriptional activity (Kim et al. 2001; Krishnan et al. 1993; Kuiper et al. 1998). However, alternate targets of BPA action have recently been identified (Bouskine et al. 2009; Takayanagi et al. 2006; Takeshita et al. 2001), and the exact mechanism by which BPA functions is currently unknown. Most of the literature has focused on the effects of short-term BPA exposure, administered during specific windows of early-life development, and the resultant later-life consequences. These studies have shown early-life exposure to BPA in female rodents alters the onset of puberty, disrupts estrous cyclicity and normal mammary gland development, and increases the development of preneoplastic lesions in the mammary gland, cell proliferation, and the incidence of mammary gland hyperplasia in transgenic mice lacking BRCA1 (Durando et al. 2007; Howdeshell et al. 1999; Jones et al. 2010; Markey et al. 2001, 2003; Munoz-de-Toro et al. 2005; Murray et al. 2007; Vandenberg et al. 2008; Wadia et al. 2007).

We have shown recently that oral BPA exposure during the prenatal period or prepubertal period accelerates carcinogenesis in a model of chemically induced mammary cancer (Betancourt et al. 2010; Jenkins et al. 2009). Alterations to cell proliferation and apoptosis in the mammary gland were accompanied by molecular changes, including increased expression and/or activation of the erbB family of receptor tyrosine kinases. These findings led us to hypothesize that a subpopulation of adults exist, namely women with HER2/erbB2-positive breast cancer, that could be negatively affected by chronic BPA exposure during adulthood. Women with breast cancer that overexpresses HER2/erbB2 account for 15–30% of all diagnosed pathology subtypes and are usually associated with an unfavorable clinical outcome (reviewed by Barros et al. 2010).

## Materials and Methods

*Chemicals and antibodies.* BPA was purchased from Sigma Chemical Company (St. Louis, MO). Antibodies to epidermal growth factor receptor (EGFR), erbB2, phosphorylatederbB3, phosphorylatederbB3, insulin-like growth factor 1 receptor (IGF-1R), phosphorylated-IGF-1R, phosphorylated-Bad, PI3K, PTEN, Akt 1, Akt 3, phosphorylatedAkt, glycogen synthase kinase-3-beta (GSK-3β), and phosphorylatedGSK-3β were purchased from Cell Signaling Technologies (Danvers, MA). Antibodies to erbB3 and IGF-1 were purchased from Santa Cruz Biotechnology (Santa Cruz, CA).

*Animal care and use.* Animal care and use were conducted according to established guidelines approved by the Institutional Animal Care and Use Committee at the University of Alabama at Birmingham. All animals were housed in a temperature-controlled facility with a 12-hr light/dark cycle. All animals were treated humanely and with regard for alleviation of suffering. A colony of mouse mammary tumor virus (MMTV)-erbB2/neu transgenic mice [FVB/N-TgN(MMTV-neu202Mul)] was established through breeding pairs purchased from Jackson Laboratory (Bar Harbor, ME). Because progesterone and pregnancy have been reported to drive the MMTV promoter, only virgin female mice were used. Animals were fed AIN-76A diet (phytoestrogen-free; Dyets, Inc., Bethlehem, PA), housed in polypropylene cages, and provided glass water bottles.

**Figure 1:**
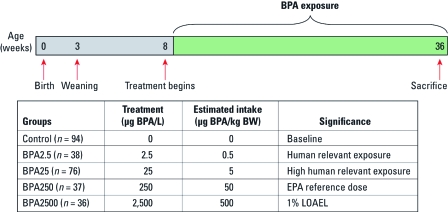
Beginning at 56 days of age, female MMTV-erbB2 mice were provided 0, 2.5, 25, 250, or 2,500 µg BPA/L drinking water. Estimated daily intakes were calculated based on a 20-g mouse drinking 4 mL water daily. This is based on our preliminary study, which found that mice drink 4 mL water daily (unpublished pilot data). LOAEL, lowest observed adverse effect level.

*Tumorigenesis.* Female MMTV-erbB2 mice were exposed to BPA via the drinking water, beginning at 56 days of age and continuing for the lifetime of the animals (252 days of age). Cagen et al. (1999) and our own preliminary data have shown BPA to be stable in water for 1 week. Water with and without BPA was supplied fresh weekly. The following treatment groups were set up: 0 (control) and 2.5 (BPA2.5), 25 (BPA25), 250 (BPA250), and 2,500 (BPA2500) µg BPA/L drinking water (Figure 1). Drinking water of all groups, including the control group, contained 0.05% by volume of the vehicle, ethanol.

All animals were palpated twice weekly. Mice were sacrificed at 252 days of age or when tumors exceeded 10% BW. At sacrifice, animals underwent complete necropsy. All tumors, gross lesions, and lung sections were dissected and blocked in paraffin. Tumor volume was calculated by measuring the length, width, and height of the dissected tumor. All dissected tumors were pathologically graded according to the consensus statement of the Annapolis meeting of medical and veterinary pathologists (Cardiff et al. 2000). Only those tumors classified as invasive mammary adenocarcinoma were used in the final analysis of tumor latency (time to first, second, and third tumor), volume, and multiplicity. Quantification of lung metastases was determined through inflating the lungs with an India ink solution [15% vol/vol India ink, 0.5% (vol/vol) ammonia solution]. Excised lesions as well as whole-lung sections were evaluated. All pathological analyses were carried out under blinded conditions by a board-certified pathologist (I.E).

*Cell proliferation index.* We selected 112 days of age as the end point for mechanistic studies; at this age, no preneoplastic/neoplastic lesions were identified in the mammary glands of untreated mice in a preliminary ontogeny study (data not shown). At 112 days of age, the number four abdominal mammary glands were collected from MMTV-erbB2 mice treated with 0, 2.5, 25, 250, and 2,500 µg BPA/L drinking water. Tissue sections were deparaffinized and rehydrated through xylene and graded alcohol washes. Slides were boiled in citrate buffer, incubated in hydrogen peroxide, and blocked using serum. The slides were incubated in Ki-67 primary antibody (Dako, Glostrup, Denmark) overnight in a humidified chamber. After incubating the tissue sections in the appropriate conjugated secondary antibody, we employed the ImmPRESS kit (Vector Laboratories, Burlingame, CA). Positively stained cells were visualized by incubating the tissue sections with 3,3´-diamonobenzidine (DAB) and counterstained with hematoxylin. Tissue sections were dehydrated with graded alcohols, cleared with xylene, and mounted with a glass coverslip. A minimum of 500 mammary epithelial cells were counted from at least five different structures. All counts were performed in duplicate under blinded conditions, with the final index for each section being a mean of the duplicate counts.

*Apoptotic index.* The index of apoptosis was determined through the use of the ApopTag Plus Peroxidase *In Situ* Apoptosis Detection kit (Chemicon International, Billerica, MA) according to the manufacturer’s protocol. Cells that stained positively and exhibited morphologic characteristics of apoptosis were counted as positive. A minimum of 500 mammary epithelial cells were counted from at least five different structures. All counts were performed in duplicate under blinded conditions, with the final index for each section being a mean of the duplicate counts. A cell proliferation to apoptosis ratio was calculated for each individual gland.

*Immunoblotting.* Mammary glands were homogenized in RIPA lysis buffer (Pierce Biotechnolgy, Rockford, IL) with the use of a pestle and grinding kit (GE Healthcare Inc., Piscataway, NJ). Protein lysate was quantified using the Bradford assay (BioRad Laboratories, Hercules, CA). Equal protein content (40 μg) was loaded onto precast SDS Tris-HCl 4–20% polyacrylamide gels (BioRad Laboratories). Proteins were transferred to a nitrocellulose membrane overnight. The membrane was blocked and incubated in primary antibody. Secondary antibody and chemilume (Pierce Biotechnology) were added, and protein expression was visualized using film. Densitometry was assessed using Quantity One (BioRad Laboratories). Positive protein controls purchased from the supplier of the corresponding antibodies and Kaleidoscope Precision Plus Protein standards (BioRad Laboratories) were employed to identify the protein of interest.

*Statistical analysis.* The statistical method for evaluating tumor latency was performed as previously described (Betancourt et al. 2010). Tumor multiplicity was analyzed using the Cochran–Armitage test. The incidence of pulmonary metastasis was compared using Fisher’s exact test. Normally distributed data were analyzed using analysis of variance followed by Bonferroni’s multiple comparison test (MCT). Data not normally distributed were analyzed using the Kruskal–Wallis test followed by Dunn’s MCT.

## Results

*Oral exposure to low BPA doses alter mammary tumorigenesis.* Beginning at 56 days of age, female MMTV-erbB2 transgenic mice were exposed to BPA2.5, BPA25, BPA250, or BPA2500 via the drinking water. These doses of BPA allowed investigation of a broad range of BPA daily intake values, ranging from doses estimated to be at human relevancy (BPA2.5 and BPA25) to exposures based on the existing regulatory daily intake limits of BPA in the United States (BPA250 and BPA2500) (Figure 1) (National Toxicology Program 1982).

**Figure 2:**
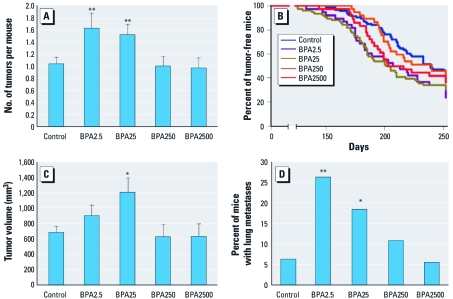
Tumor multiplicity (mean ± SE) (*A*), time-to-first-tumor latency as calculated from birth (*B*), tumor volume (mean ± SE) (*C*), and incidence of pulmonary metastasis (*D*) in MMTV-erbB2 transgenic mice after chronic oral intake of BPA through the drinking water. In *B*, *p* = 0.02 for BPA2.5, and *p* = 0.01 for BPA25, both compared with control. *p ≤ 0.05, and **p ≤ 0.01 compared with control.

Chronic consumption of low doses of BPA significantly accelerated several end points of mammary tumorigenesis in female MMTV-erbB2 transgenic mice. Both BPA2.5 (*p* = 0.01) and BPA25 (*p* = 0.01) significantly increased tumor multiplicity, increasing the mean number of mammary tumors by 58% and 47%, respectively, compared with control (Figure 2A). The low doses of BPA also significantly reduced the median time to first tumor onset by at least 29 days compared with control (Figure 2B). The mean tumor volume was also significantly increased after chronic exposure to BPA25 (*p* < 0.05) and trended toward an increase with BPA2.5 treatment (Figure 2C). Further, chronic exposure to low doses of BPA significantly increased the number of mice that developed pulmonary metastasis (Figure 2D). All of these effects were absent in the higher, regulatory-based doses of BPA. No significant differences between treatments were noted for the location, incidence of necrosis, or the grade of the developing tumor (data not shown).

*Overt markers of toxicity.* Only BPA250 significantly altered BW (Table 1). This alteration represents a loss in BW of < 10% compared with control; this BW loss failed to reach toxicological significance. Chronic BPA exposure also caused a slight increase in uterine wet weight and uterine wet weight to BW ratio, with BPA250 achieving statistical significance (Table 1).

**Table 1:** The effects of chronic oral BPA on body and uterine weights.

Treatment	BW (g)	Uterine weight (mg)	Uterine:BW ratio
Control		26.0 ± 0.3		90.2 ± 4.3		3.5 ± 0.2
BPA2.5		25.3 ± 0.6		100.7 ± 7.2		4.0 ± 0.3
BPA25		25.9 ± 0.4		107.3 ± 6.0		4.2 ± 0.2
BPA250		24.1 ± 0.2*		123.3 ± 13.6*		5.1 ± 0.6^#^
BPA2500		24.4 ± 0.3		113.0 ± 6.5		4.6 ± 0.3
BW, uterine wet weight, and uterine-to-BW ratio in 36-week-old MMTV-erbB2 mice after chronic oral exposure to BPA via drinking water. Values represent mean ± SE. *p ≤ 0.05, and ^#^*p *≤ 0.001 compared with control.						

**Figure 3:**
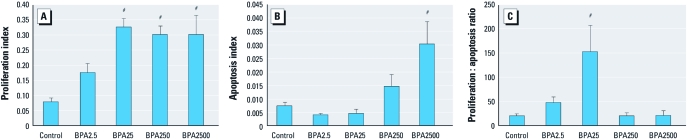
Proliferation index (*A*), apoptotic index (*B*), and cell proliferation-to-apoptosis ratio (*C*) in mammary gland epithelial cells of 112-day-old MMTV-erbB2 mice treated ± chronic, oral BPA (*n *= 5–17 mice/treatment) via the drinking water. Values represent mean ± SE. ^#^*p* ≤ 0.001 compared with control.

*Cellular proliferation and apoptosis in the mammary gland in response to BPA.* At 112 days of age, MMTV-erbB2 mice exposed to low BPA doses (BPA2.5 and BPA25) exhibited a dose-dependent increase in the cell proliferation index in mammary gland epithelial cells before plateauing at the higher doses (Figure 3A). BPA exposure also caused a dose-dependent increase in the apoptotic index in mammary epithelial cells (Figure 3B). This resulted in the highest administered dose, BPA2500, having a significantly greater apoptotic index compared with control. When cell proliferation and apoptosis were taken together to estimate cell turnover in mammary epithelial cells, the resultant cell proliferation to apoptosis ratio produced a nonlinear dose–response curve that closely mimicked the tumorigenic response of MMTV-erbB2 mice after chronic exposure to BPA (Figure 3C). Both BPA2.5 and BPA25 resulted in cell proliferation to apoptosis ratios greater than control, with BPA25 achieving statistical significance. The higher doses of BPA administered in this study, BPA250 and BPA2500, did not differ significantly from control (Figure 3C).

**Figure 4:**
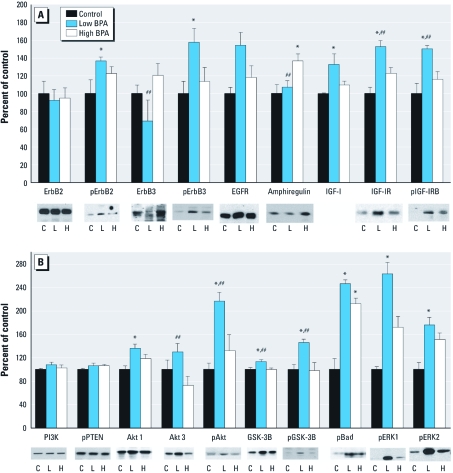
(*A*) Protein expression and/or phosphorylation of erbB2, phosphorylated-erbB2, erbB3, phosphorylated-erbB3, EGFR, amphiregulin, IGF-1, IGF-1R, and phosphorylated-IGF-1R in the mammary glands of MMTV-erbB2 mice exposed to 0 (control), 25 (low BPA), or 2,500 (high BPA) µg BPA/L drinking water (*n* =**6–8 mice/treatment). (*B*) Protein expression and/or phosphorylation of PI3K, PTEN, Akt 1, Akt 3, phosphorylated-Akt, GSK-3β, phosphorylated-GSK-3β, phosphorylated-Bad, phosphorylated-ERK1, and phosphorylated-ERK2 in the mammary glands. Graphs represent mean density ± SE as a percentage of the control. Representative blots for each of the proteins analyzed by immunoblots are included under the graphs (IGF-1 is excluded because it was quantitated via ELISA). For blot images, C, L, and H indicate representative images from control, low BPA, and high BPA samples, respectively. *≤ 0.05 compared with control; ^##^≤ 0.05 compared with BPA2500.

*Chronic BPA exposure alters the protein expression of signaling pathways involved in carcinogenesis.* To determine whether oral exposure to BPA can interfere with the erbB and associated signaling pathways, we performed targeted immunoblot analysis using mammary glands from 112-day-old MMTV-erbB2 mice exposed to representative doses of BPA (Figure 4). BPA25 was selected from the low BPA doses because it altered all end points of tumorigenesis and resulted in the greatest disturbance of cell turnover compared with control. BPA2500 was selected from the high BPA doses because, despite not altering mammary tumorigenesis, it exhibited significantly increased indices of cell proliferation and apoptosis.

To ensure that BPA did not accelerate tumorigenesis through driving the expression of the transgene, we first investigated the expression of erbB2 (Figure 4A). No differences were observed in the protein expression of erbB2 between any of the treatment groups. Despite this, exposure to BPA25, but not BPA2500, significantly increased the phosphorylation of erbB2 compared with control. Although BPA25 significantly decreased the expression of erbB3 compared with BPA2500-treated mice, it significantly increased erbB3 phosphorylation compared with control. The protein expression of EGFR (also known as erbB1) was not altered by any of the treatment groups. Measuring the protein expression of the erbB ligand reported to be under the control of ER action, amphiregulin, we found a dose-dependent increase in protein expression. Accordingly, BPA2500 exhibited a significant increase in the expression of amphiregulin compared with control and BPA25. Chronic exposure to BPA25 significantly up-regulated the expression and phosphorylation of IGF-1R compared with control and BPA2500. We investigated the local expression of IGF-1R ligand, IGF-1, in the mammary gland as opposed to circulating values found in the serum. BPA25 treatment significantly increased the local mammary gland expression of IGF-1 ligand compared with control.

Next, we assessed the downstream Akt signaling pathway (Figure 4B). The expression of PI3K and PTEN, which function as opposing gatekeepers for the activation of Akt, did not differ significantly between any of the treatment groups at 112 days of age. BPA25 treatment significantly increased the expression of the Akt 1 isoform compared with control and the Akt 3 isoform compared with BPA2500. Activation of Akt (pan-activation), as measured by phosphorylated Akt, was significantly up-regulated by BPA25 compared with control and BPA2500.

In addition, several downstream proteins involved in this pathway were regulated, including GSK-3β, Bad, and the extracellular signal-regulated kinases (ERKs 1 and 2). Normally involved in the inhibition of glycogen synthesis and tagging the oncogenic beta catenin for degradation, GSK-3β is phosphorylated and consequently inactivated by Akt. BPA25 significantly increased the phosphorylation of GSK-3β (inactive) compared with control and BPA2500. A slight, although statistically significant, increase in total GSK-3β protein expression was also noted in response to BPA25 compared with control and BPA2500. Bad, which normally functions as a pro-apoptotic protein, also becomes inactivated when phosphorylated by Akt. BPA25 and BPA2500 significantly up-regulated the phosphorylation (inactivation) of Bad compared with control. The ERKs are involved in downstream signaling initiated by both the erbB and IGF family of receptor tyrosine kinases and have been shown to play integral roles in cellular proliferation. The phosphorylation of ERK1 and ERK2 was up-regulated in response to BPA25, but not BPA2500, treatment.

## Discussion

Despite several countries altering regulations governing BPA intake in humans, limitations in the United States have remained largely unchanged. This has been blamed on a lack of compelling evidence that BPA is capable of causing harm (European Food Safety Authority 2006; Food and Drug Administration 2008), citing study limitations in the existing literature such as using inappropriate routes of administration, using a concentration range of doses that is too narrow, pursing end points not directly tied to pathology, and using statistically unsound sample sizes. Thus, the aim of this study was to evaluate the impact of chronic, oral BPA exposure during adulthood on the development and progression of mammary carcinogenesis while attempting to address some of the aforementioned deficits.

The doses of BPA administered in this study were selected with human applicability in mind. Recent studies suggest that the average adult is exposed to low concentrations of BPA, no more than 0.4–1.5 µg BPA/kg BW per day (FAO/WHO 2010; Lakind and Naiman 2008). Further, the 95th percentile of BPA exposure does not likely exceed 1.5–4.2 µg BPA/kg BW per day. Our mice were exposed to 0, 2.5, 25, 250, or 2,500 µg BPA/L drinking water. Preliminary studies established a mean BW of 20 g and a daily intake of approximately 4 mL fluid. Thus, our doses resulted in the estimated daily consumption of 0.5, 5, 50, and 500 µg BPA/kg BW, respectively. These doses of BPA allowed investigation of a broad range of BPA intake values, ranging from doses estimated to be of human relevancy (BPA2.5 and BPA25) to exposures based on daily regulatory intake limits of BPA in the United States (BPA250 and BPA2500). Future efforts should focus on validating these estimated intake values by measuring circulating serum BPA values.

Chronic administration of BPA resulted in a nonmonotonic dose response for many of the tumorigenesis end points, with human-relevant concentrations of BPA (BPA2.5 and BPA25) decreasing tumor latency and increasing tumor multiplicity, tumor volume, and the incidence of metastasis. Although tumor grade did not differ significantly between treatment groups, significant increases in tumor volume and the incidence of metastasis in mice chronically consuming human-relevant concentrations of BPA support the recent study by Dairkee et al. (2008), which suggested that BPA may increase aggressive tumor formation. Furthermore, the regulatory-based concentrations (BPA250 and BPA2500) of BPA did not significantly alter any end point of the tumorigenesis study. These data suggest that chronic BPA consumption during adulthood in MMTV-erbB2 mice may function in a nonlinear fashion for the development and progression of mammary carcinogenesis. In this model, lower concentrations of BPA acted more potently than higher doses. Whether this pattern holds true in other animal models is not known and strongly warrants further investigation.

However, nonmonotonic dose–response curves have been noted previously in the literature. Hugo et al. (2008) found that fat explants treated with BPA (0.1–10 nM) induced a U-shaped curve of adiponectin secretion, with low doses inhibiting and higher doses increasing adiponectin secretion. Using IRC mice, Miyawaki et al. (2007) administered 1 or 10 µg BPA/L drinking water during perinatal development. At the time of sacrifice, 1 µg, but not 10 µg, BPA/L significantly increased adipose tissue weight and the concentration of circulating serum leptin (Miyawaki et al. 2007). Considering that BPA has been described as a xenoestrogen, it is interesting to note that Vandenberg et al. (2006) found that mice exposed to estradiol exhibited a nonmonotonic dose response for mammary gland morphology. Other classes of compounds, most notably the angiogenesis inhibitors, have also reported nonmonotonic therapeutic dose responses (Benelli et al. 2003; Bruns et al. 2004; Celik et al. 2005; Humar et al. 2002; Lalani et al. 2004; Motegi et al. 2002; Panigrahy et al. 2002; Slaton et al. 1999; Tjin Tham Sjin et al. 2005, 2006; Trinh et al. 2000; Yamaguchi et al. 1999). Given that our data suggest a potential role for BPA in tumor angiogenesis because of the significant increases in tumor volume and the incidence of metastasis, future research efforts focusing on this connection are warranted.

We focused our attention on investigating potential mechanisms behind the nonmonotonic dose response in the MMTV-erbB2 mouse. At the cellular level, chronic BPA administration during adulthood caused dose-dependent increases in cell proliferation and apoptosis indices in the mammary gland. Several studies investigating early-life exposure to BPA have reported similar results (Betancourt et al. 2010; Durando et al. 2007; Jenkins et al. 2009; Munoz-de-Toro et al. 2005; Murray et al. 2007; Vandenberg et al. 2008). When the cell proliferation indices and apoptotic indices were combined to produce an estimation of cell turnover in the mammary gland, a nonmonotonic dose–response curve resulted. This nonmonotonic dose–response curve was the best predictor of the ability of each concentration of BPA to alter tumorigenesis.

Our data suggest that the nonmonotonic dose response observed for tumorigenesis is due, at least in part, to the differing ability of each concentration of BPA to induce apoptosis. All of the BPA doses studied were capable of increasing the cell proliferation index. However, only the higher doses of BPA were capable of countering the increased cell proliferation with a simultaneous increase in apoptosis in mammary gland epithelial cells. As a result, neither of the regulatory-based doses produced a ratio of cell turnover in the mammary gland that differed from controls. Conversely, without an increase in apoptosis, the lower doses of BPA resulted in a mammary gland where increased cell proliferation was coupled to decreased apoptosis, skewing normal cell turnover and potentially contributing to carcinogenesis.

Our next aim was to determine which signaling pathways may contribute to the ability of BPA to alter tumorigenesis. Using a representative treatment group from each category of exposure, human relevant and regulatory based, we report distinct differences in protein expression and activation. The MMTV-erbB2 transgenic mouse model is driven by the aberrant expression of the erbB2 growth factor receptor (Guy et al. 1992). Overexpression of other growth factor receptors, such as erbB3 and IGF-1, and the downstream Akt pathway has also been reported to occur or contribute to carcinogenesis in this model (Kim et al. 2005; Pape-Ansorge et al. 2002; Siegel et al. 1999).

Neither low nor high exposure to BPA altered the protein expression of the transgene erbB2. However, the low BPA dose significantly increased the phosphorylation of erbB2 and erbB3, the expression and phosphorylation of the IGF-1R, and the local expression of IGF-1. The higher BPA dose did not significantly alter either the expression or phosphorylation of any of the receptors or ligands. The downstream signaling pathways of erbB2 are largely dictated by its receptor dimerization partner. The erbB3 receptor contains six docking sites for PIP3, which serves as a docking site of the serine kinase Akt. Heterodimerization between erbB2 and erbB3 has been reported to preferentially induce the activation of the Akt pathway. Thus, with BPA25 significantly increasing erbB2 and erbB3 phosphorylation, it was not surprising to find that it also increased Akt isoform expression and phosphorylation. Again, these effects were absent in the BPA2500 treatment group. Collectively, our results suggest biologically distinct mechanisms for BPA that differ according to the dose administered. Further, the data presented here suggest that aberrant signaling in the erbB and IGF signaling pathways may play a role in the detrimental effects of low-dose BPA.

## Conclusions

The data presented here provide the first evidence that oral administration of BPA affects cancer development and progression in a nonmonotonic fashion. Our research supports that differing doses of BPA may function in distinctly different, sometimes opposing, manners at both the tissue and molecular level. That these data are derived from a genetically distinct model (MMTV-erbB2 mouse) may be a compelling concern for a population at risk, that is, women with HER2/neu overexpressing breast cancer, who account for 15–30% of all women diagnosed with breast cancer. Although no model is a perfect predictor of human disease, the MMTV-erbB2 mice were selected over comparable models for several reasons. Most important, this provides a model that exhibits *a*) histopathological development that is similar to humans, *b*) clinically relevant molecular cancer subtype, *c*) lengthy tumor latency, *d*) low tumor multiplicity, and *e*) high incidence of pulmonary metastasis (Di Carlo et al. 1999; Guy et al. 1992). Although data obtained in animals may not translate perfectly to humans, the data presented here do cause some concern for the current conditions of BPA use, exposure, and regulation. Future research should pursue whether these findings can be replicated in other modeling systems of differing tumor pathologies.
